# Acute myeloid leukemia post‑cytotoxic therapy following chemotherapy for thymoma: A case report

**DOI:** 10.3892/mi.2024.133

**Published:** 2024-01-11

**Authors:** Masahiro Manabe, Yoko Tani, Naoyuki Inano, Yuuji Hagiwara, Nobuhiro Sogabe, Satoru Nanno, Ki-Rhang Koh

**Affiliations:** 1 Department of Hematology, Osaka General Hospital of West Japan Railway Company, Osaka 545-0053, Japan; 2 Department of Clinical Oncology, Osaka Metropolitan University Graduate School of Medicine, Osaka 545-8585, Japan; 3 Department of Clinical Laboratory, Osaka General Hospital of West Japan Railway Company, Osaka 545-0053, Japan

**Keywords:** thymoma, acute myeloid leukemia, chemotherapy, therapy-related, harlequin cell

## Abstract

The present study reports the case of a patient with acute myeloid leukemia post-cytotoxic therapy (AML-pCT) that developed following chemotherapy for thymoma. A 64-year-old female patient underwent surgical resection for a mediastinal tumor and was diagnosed with stage IVa thymoma. She received chemotherapy, including carboplatin/etoposide, carboplatin/paclitaxel and amrubicin monotherapy. At 56 months following surgery, she developed blastosis and was diagnosed with AML-pCT. As demonstrated herein, although treatment for thymoma is associated with a markedly lower frequency of myeloid neoplasms post-cytotoxic therapy (MN-pCT) than treatment for other malignancies, such as breast carcinoma, it is important to be aware that MN-pCT may occur as a late complication of thymoma treatment.

## Introduction

Chemotherapy and radiotherapy have contributed to improvements in the clinical outcomes of cancer patients. However, secondary malignancy is one of the most critical late complications associated with cytotoxic treatment. Although myeloid neoplasms post-cytotoxic therapy (MN-pCT), such as acute myeloid leukemia (AML) and myelodysplastic syndrome (MDS) are well-known complications in the sphere of hematology (1), they have rarely been reported in patients with thymoma.

The present study describes the case of a patient with AML post-cytotoxic therapy (AML-pCT) that developed following chemotherapy involving carboplatin/etoposide, carboplatin/paclitaxel and amrubicin monotherapy for thymoma.

## Case report

A 64-year-old female patient was referred to the Osaka Metropolitan University Hospital (Osaka, Japan) due to an abnormal shadow on a chest X-ray. A computed tomography scan revealed an anterior mediastinal mass measuring 6x7 cm. The patient underwent surgical resection, and a pathological examination of a resected specimen revealed thymoma, type B1. Pleural metastases and malignant pleural effusion were detected during surgery; therefore, the patient was diagnosed with stage IVa disease (T3N0M1a, according to the Union for International Cancer Control classification) (2). The patient underwent post-operative surveillance, and computed tomography performed at 16 months post-surgery revealed disease recurrence. Thereafter, the patient received four cycles of chemotherapy involving carboplatin and etoposide. Although the recurrent tumor yielded a partial response, pleural metastases indicated regrowth at 25 months following surgery. At this time, widespread multiple pleural metastases were detected; hence, it was considered that surgical treatment and radiotherapy were not suitable for this case due to the impossibility of achieving complete surgical treatment and tumor control by radiotherapy on the basis of the pre-operative evaluation. A total of four cycles of second-line chemotherapy, including carboplatin and paclitaxel resulted in progressive disease. Therefore, third-line chemotherapy (amrubicin monotherapy) was administered. Although the patient achieved a partial response, blasts in peripheral blood were detected in the 27th cycle. The blasts in peripheral blood gradually increased to >20% and, thus, the patient was referred to the Department of Hematology (Osaka General Hospital of West Japan Railway Company). A bone marrow examination was performed, which revealed an excess of blasts. Based on this finding, the patient was diagnosed with AML-pCT at 56 months following surgery.

**Figure 1:**
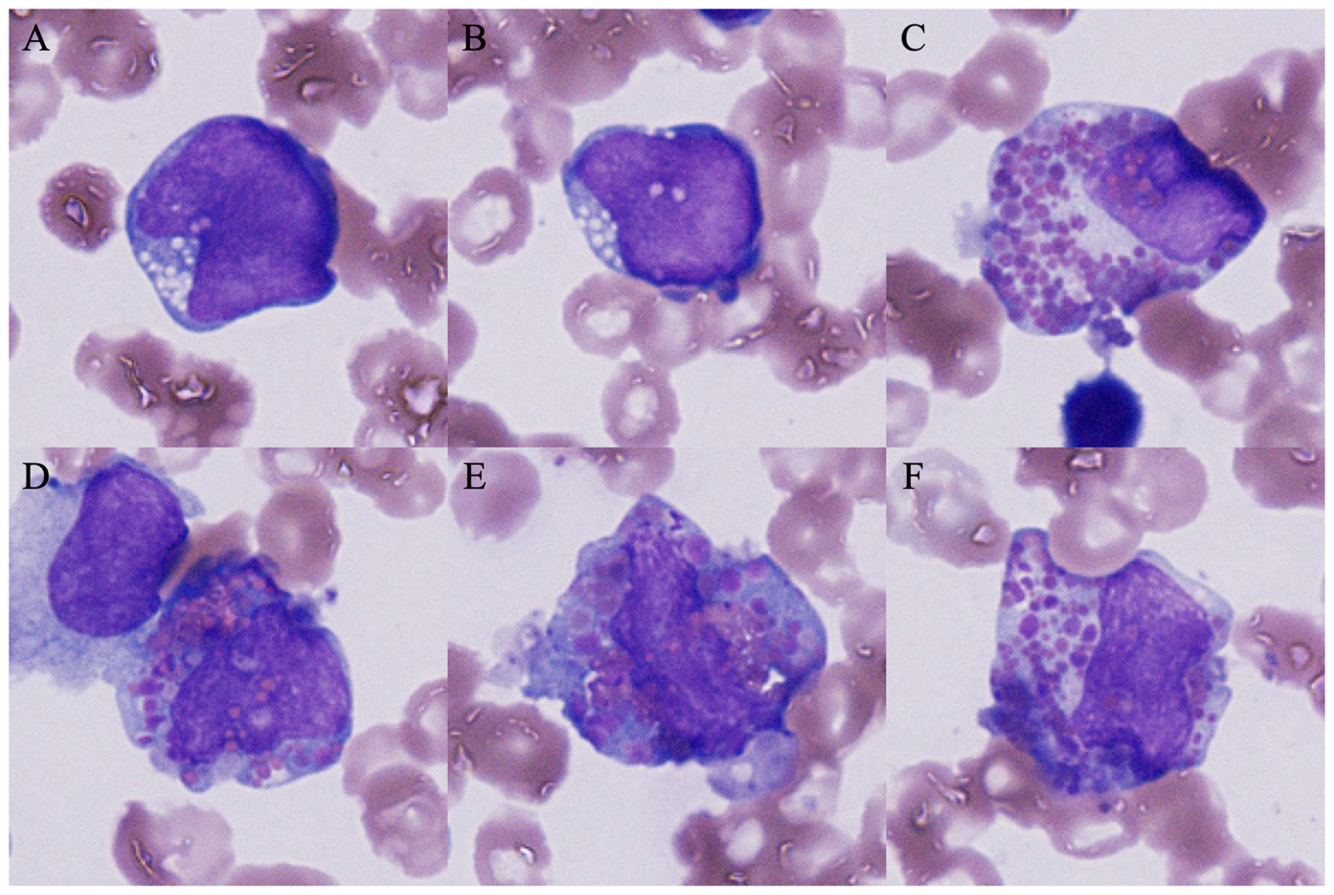
Bone marrow cells at the relapse of leukemia (May-Giemsa staining, original magnification, x1,000). Blasts harboring (A and B) fine chromatin and (C-F) the so-called harlequin cells were observed.

**Figure 2:**
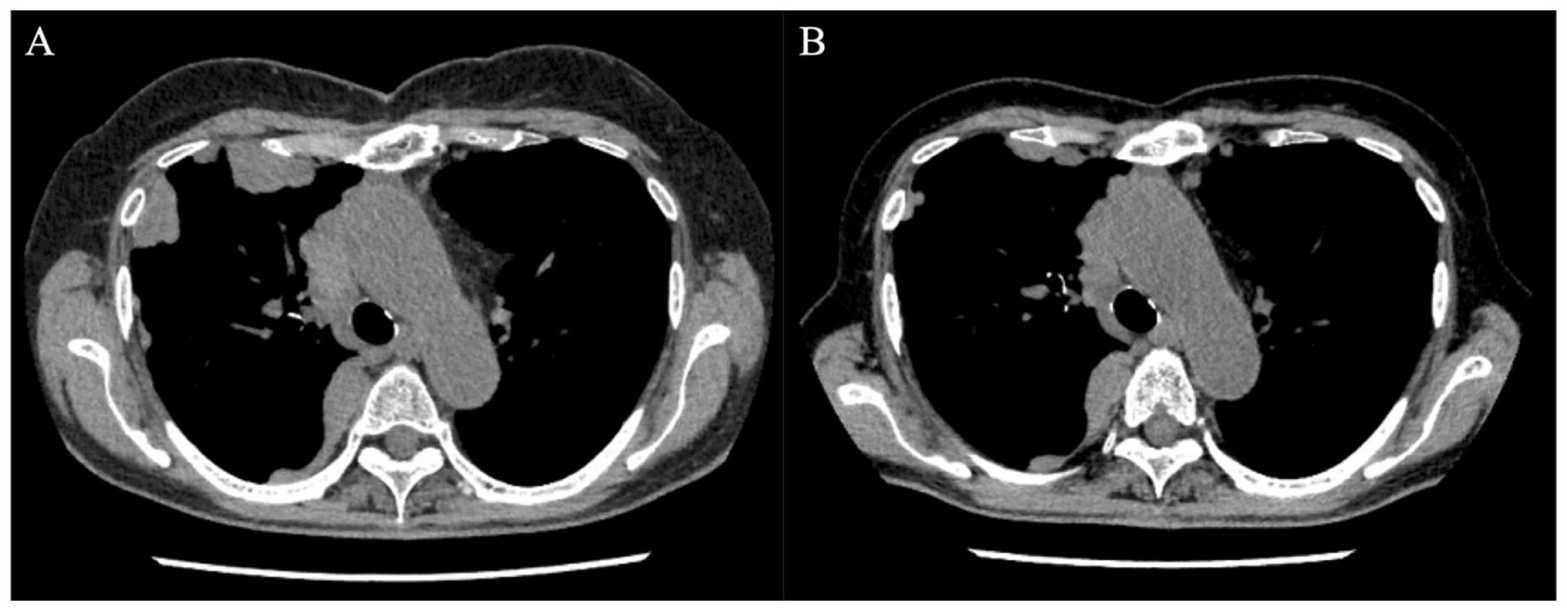
Computed tomography illustrating (A) multiple pleural masses, and (B) following chemotherapy involving idarubicin and cytosine arabinoside, the pleural masses exhibited an improvement.

A chromosomal analysis of the bone marrow cells revealed the following unbalanced complex karyotype: 46,XX,add(6)(q23)[1]/46,idem,-19,+mar[1]/47,XX,+mar1[6]/46,XX[11]. The patient received induction chemotherapy involving daunorubicin and cytosine arabinoside, and achieved complete remission. Thereafter, three cycles of consolidative chemotherapy consisting of high-dose cytosine arabinoside were administered. However, the regrowth of pleural metastases of thymoma was observed. A total of ten cycles of amrubicin monotherapy were attempted; however, these resulted in progressive disease. AML-pCT also recurred at 55 months following surgery. Although the patient received six cycles of azacitidine monotherapy, pancytopenia gradually progressed. At this time, a bone marrow examination revealed an excess of blasts (31.6%). In addition, some blasts harboring both eosinophilic and basophilic granules in their cytoplasm, so-called harlequin cells, were detected (Fig. 1). The cells were fixed with absolute methanol for 10 min, stained with May Grünwald solution (cat. no. 954-0791-7; Sysmex Corporation) for 5 min, stained with Giemsa solution (cat. no. 954-0801-7; Sysmex Corporation) for 5 min and then in buffer (pH 6.8) for 2 min, all at room temperature. Images were captured using a light microscope (BX43, Olympus Corporation) equipped with a DP73 camera (Olympus Corporation). The patient began induction chemotherapy with idarubicin and cytosine arabinoside. Although the residual lesions of metastatic thymoma exhibited improvement (Fig. 2), the remission of leukemia was not achieved. A total of three cycles of chemotherapy involving azacitidine and venetoclax, and re-induction chemotherapy with etoposide, cytosine arabinoside and mitoxantrone were attempted; however, this resulted in leukemic progression. The patient opted to discontinue chemotherapy and was discharged from the hospital at 102 months following surgery.

## Discussion

MN-pCT are some of the most common secondary malignancies occurring as late complications of cytotoxic therapy. MN-pCT may be caused by conventional chemotherapy, as well as large-field radiation therapy. AML-pCT accounts for 7-8% of all AML cases (3-5). The association between the causative treatment and leukemogenesis differs for alkylating agents and topoisomerase II inhibitors. Alkylating drugs frequently cause deletion-type chromosomal abnormalities, such as monosomy and/or the deletion of chromosomes 5 and 7, and AML develops through MDS 5 to 7 years after their administration (6). Furthermore, platinum agents, such as carboplatin, which was administered to the patient described in the present study, act as alkylators. On the other hand, topoisomerase II inhibitors, including anthracycline, cause AML to develop within 2 years of their administration without undergoing MDS, and are often accompanied by balanced chromosomal translocations (6). Amrubicin and etoposide administered to the patient in the present study were applicable to this category, and a platinum agent harboring an alkylating effect appeared to be the causative drug as AML occurred >2 years following the initiation of chemotherapy for thymoma, in addition to the presence of unbalanced chromosomal abnormalities at its diagnosis.

The most common cause of AML-pCT is breast cancer, followed by hematological malignancies, and thyroid, gastrointestinal, prostate and testicular cancer (3). However, the incidence of MN-pCT that develop after other malignancies, such as ovarian cancer and skin cancers, is low. Furthermore, cases of MN-pCT in which thymoma is the primary disease are even rarer. As regards the disease incidence, Kayser *et al* (3) detected only 2 patients (1%) who received cytotoxic therapy for mediastinal cancers among 200 patients with AML-pCT. Although there have been cases of AML-pCT occurring in patients with thymic cancer receiving long-term chemotherapy (7), such as case described herein, they are extremely rare. Apart from therapy-related complications, thymoma is associated with secondary malignancies, and leukemia has also been reported (8), although at a low incidence rate. Although it is unclear whether the scarcity of MN-pCT cases caused by treatment for thymoma is due to these cases truly being rare or misdiagnosed (i.e., as *de novo* myeloid neoplasm or secondary malignancy associated with thymoma), the accumulation of further cases is required in order to obtain accurate data on the frequency of MN-pCT following treatment for thymoma.

As regards clinical outcomes, the prognosis of MN-pCT, including overall survival, is poorer than that of *de novo* AML; i.e., the estimated median overall survival is 10-14 months (3,5,9). However, the therapeutic outcomes of patients with thymoma have recently improved due to advances in surgery and chemotherapy. In addition, the median latency period between the diagnosis of the primary malignancy and the occurrence of AML-pCT was 4.04 years (3). As the incidence of MN-pCT in patients with thymoma may increase in the future as treatment advances prolong their survival, as has been observed in all cancer patients in the USA (10), it is critical for clinicians in thoracic surgery/clinical oncology departments to be aware that MN-pCT may occur as a late complication of treatment for thymoma.

Concerning the treatment outcome of the case described in the present study, chemotherapy for AML resulted in a refractory course. On the other hand, chemotherapy involving idarubicin and cytosine arabinoside contributed to a reduction in the residual pleural metastases of the thymoma. The treatment of cancer patients with synchronous multiple primaries is generally challenging and often a therapeutic dilemma. Additionally, in the case of advanced disease, the selection of antitumor therapy is often difficult and mostly not based on evidence from the literature and clinical trials (11). Therefore, in cases involving refractory co-existing malignancies, the continuation of definitive chemotherapy requires careful consideration. Key points that need to be considered with synchronous malignancies are patient fitness, the prognosis of each cancer, the chance of a cure, whether one tumor may be treated radically and the other sequentially, anticipated complications, obstructive symptoms and if other similar treatments may be used (12). In addition to the performance status being good in the case present herein, cytopenia caused by AML progressed; therefore, chemotherapy for AML was prioritized following a multi-disciplinary consultation board. Although idarubicin, which is categorized as an anthracycline similar to amrubicin and was used as chemotherapy for thymoma, may have been effective, there have been no reports of similar cases, at least to the best of our knowledge. Therefore, the clinical course of the present case is considered to be valuable as a reference for future practice.

This case was morphologically characteristic in that harlequin cells were noted. Harlequin cells are defined as involving both basophilic and eosinophilic granules (13). As regards their morphological significance, harlequin cells are often observed in leukemia, particularly in AML with inv(16)(p13.1q22) or t(16;16)(p13.1;q22) (13). On the other hand, harlequin cells have also been ubiquitously detected and are not limited to hematological malignancies (14), and their evaluation remains controversial. Confirmation in large-scale studies on a large number of patients is required in order to establish whether this is common in AML-pCT, as in the case described in the present study.

Lastly, as for therapeutic strategy for the recurrence of thymoma in the patient described herein, systemic chemotherapy rather than surgical treatment and/or radiotherapy was selected. Although long-term survival was observed in a previous study following the surgical treatment of thymoma recurrences, that study examined patients with stage I-III disease and did not include cases with advanced-stage IV disease, such as in the present study (15). In addition, surgical treatment was not considered to be curable due to the large number of metastases in the case in the present study. Furthermore, radiation therapy was avoided for similar reasons. As the results of previous studies analyzing therapeutic approaches for the treatment of recurrences of thymoma have been controversial (16,17), further studies are warranted to establish which modalities among chemotherapy, surgical treatment, or radiotherapy, are appropriate for the treatment of recurrent thymoma.

## Data Availability

The datasets used and/or analyzed during the present study are available from the corresponding author upon reasonable request.
